# *cis*-Bis(2,2′-bipyridine-κ^2^ *N*,*N*′)bis­(pyridin-4-amine-κ*N* ^1^)ruthenium(II) bis­(hexa­fluoridophosphate) acetonitrile monosolvate^1^

**DOI:** 10.1107/S1600536812052002

**Published:** 2013-01-09

**Authors:** Mariana R. Camilo, Felipe T. Martins, Valéria R. S. Malta, Javier Ellena, Rose M. Carlos

**Affiliations:** a Universidade Federal de São Carlos, Departamento de Química, CP 676, CEP 13565-905, São Carlos/SP, Brazil; b Universidade Federal de Goias, Instituto de Química, Campus Samambaia, CP 131, CEP 74001-970, Goiania/GO, Brazil; c Universidade Federal de Alagoas, Centro de Ciências Exatas e Naturais, Departamento de Química, CEP 57072-970, Maceió/AL, Brazil; d Universidade de São Paulo, Instituto de Física de Sao Carlos, CP 369, CEP 13560-970, São Carlos/SP, Brazil

## Abstract

In the title complex, [Ru(C_10_H_8_N_2_)_2_(C_5_H_6_N_2_)_2_](PF_6_)_2_·CH_3_CN, the Ru^II^ atom is bonded to two α-diimine ligands, *viz.* 2,2′-bipyridine, in a *cis* configuration and to two 4-amino­pyridine (4Apy) ligands in the expected distorted octa­hedral configuration. The compound is isostructural with [Ru(C_10_H_8_N_2_)_2_(C_5_H_6_N_2_)_2_](ClO_4_)_2_·CH_3_CN [Duan *et al.* (1999 ▶). *J. Coord. Chem.* **46**, 301–312] and both structures are stabilized by classical hydrogen bonds between 4Apy ligands as donors and counter-ions and acetonitrile solvent mol­ecules as acceptors. Indeed, N—H⋯F inter­actions give rise to an inter­molecularly locked assembly of two centrosymmetric complex mol­ecules and two PF_6_^−^ counter-ions, which can be considered as the building units of both crystal architectures. The building blocks are connected to one another through hydrogen bonds between 4Apy and the connecting pieces made up of two centrosymmetric motifs with PF_6_^−^ ions and acetonitrile mol­ecules, giving rise to ribbons running parallel to [011]. 2_1_-Screw-axis-related complex mol­ecules and PF_6_^−^ counter-ions alternate in helical chains formed along the *a* axis by means of these contacts.

## Related literature

For compounds with similar properties, see: Stoyanov *et al.* (2002 ▶); Duan *et al.* (1999 ▶); Salassa *et al.* (2009 ▶). For use of 4Apy, see: Sinha & Shrivastava (2012 ▶). For the synthesis of the starting materials, see: Bonneson *et al.* (1983 ▶).
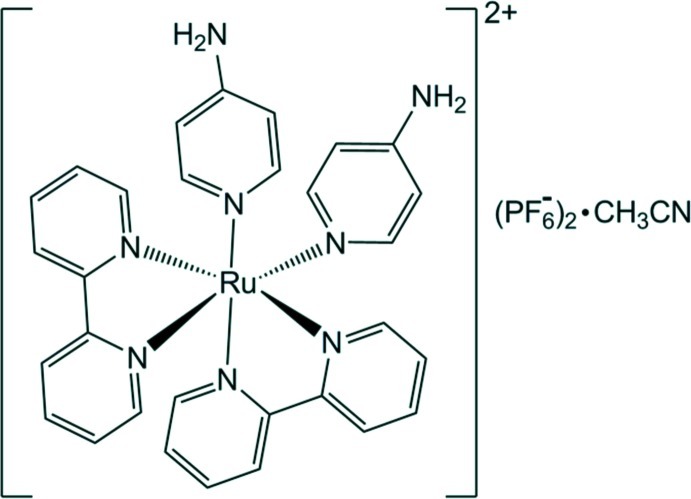


## Experimental

### 

#### Crystal data

[Ru(C_10_H_8_N_2_)_2_(C_5_H_6_N_2_)_2_](PF_6_)_2_·C_2_H_3_N
*M* *_r_* = 932.67
Triclinic, 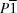

*a* = 10.8290 (3) Å
*b* = 11.8890 (3) Å
*c* = 16.1020 (4) Å
α = 104.073 (1)°
β = 99.114 (2)°
γ = 107.761 (2)°
*V* = 1853.32 (8) Å^3^
*Z* = 2
Mo *K*α radiation
μ = 0.61 mm^−1^
*T* = 298 K
0.21 × 0.11 × 0.09 mm

#### Data collection

Nonius KappaCCD diffractometer
Absorption correction: multi-scan (Blessing, 1995 ▶) *T*_min_ = 0.935, *T*_max_ = 0.950
14506 measured reflections
7906 independent reflections
6142 reflections with *I* > 2σ(*I*)
*R*_int_ = 0.033

#### Refinement

*R*[*F* ^2^ > 2σ(*F* ^2^)] = 0.053
*wR*(*F* ^2^) = 0.148
*S* = 1.06
7906 reflections
506 parameters
H-atom parameters constrained
Δρ_max_ = 0.90 e Å^−3^
Δρ_min_ = −0.58 e Å^−3^

### 

Data collection: *COLLECT* (Nonius, 2000 ▶); cell refinement: *SCALEPACK* (Otwinowski & Minor, 1997 ▶); data reduction: *DENZO* (Otwinowski & Minor, 1997 ▶) and *SCALEPACK*; program(s) used to solve structure: *SHELXS97* (Sheldrick, 2008 ▶); program(s) used to refine structure: *SHELXL97* (Sheldrick, 2008 ▶); molecular graphics: *ORTEP-3 for Windows* (Farrugia, 2012 ▶) and *Mercury* (Macrae *et al.*, 2006 ▶); software used to prepare material for publication: *WinGX* (Farrugia, 2012 ▶).

## Supplementary Material

Crystal structure: contains datablock(s) global, I. DOI: 10.1107/S1600536812052002/bg2496sup1.cif

Structure factors: contains datablock(s) I. DOI: 10.1107/S1600536812052002/bg2496Isup2.hkl

Additional supplementary materials:  crystallographic information; 3D view; checkCIF report

## Figures and Tables

**Table 1:** Hydrogen-bond geometry (Å, °)

*D*—H⋯*A*	*D*—H	H⋯*A*	*D*⋯*A*	*D*—H⋯*A*
N4—H4*A*⋯F4^i^	0.86	2.19	2.871 (8)	135
N4—H4*B*⋯F6*A* ^ii^	0.86	2.47	2.962 (7)	117
N4*A*—H4*A*1⋯F5^iii^	0.86	2.44	3.184 (8)	145
N4*A*—H4*A*2⋯N1*S* ^iv^	0.86	2.34	3.162 (13)	161

Symmetry codes: (i) 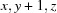; (ii) 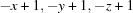; (iii) 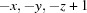; (iv) 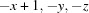.

**Fig. 1.**
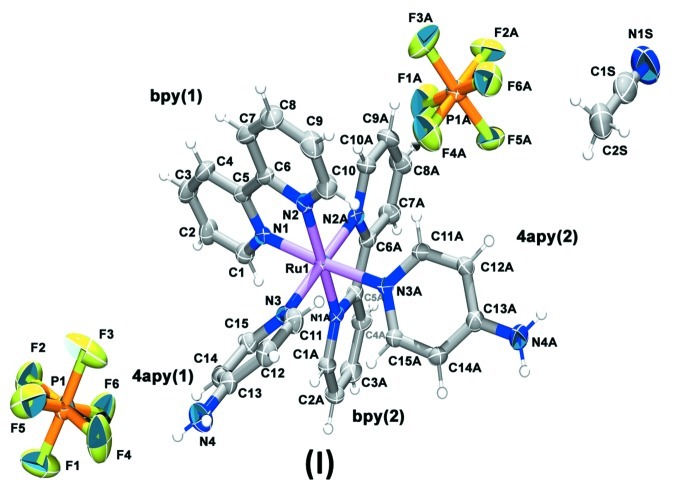
ORTEP-3 drawing of (I). Ellipsoids for non-H atoms are drawn at the 30% probability level.

**Fig. 2.**
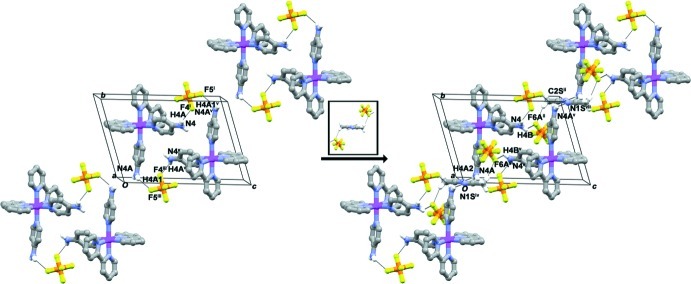
The crystal packing of (I). In this figure, three building units of (I) are shown on the left of the panel, which are joined together on the right along the [011] direction through hydrogen bonds with the connecting pieces (detached on the box). Hydrogen bonds are shown as dashed lines. [Symmetry codes: (a) (i) x, -y + 3/2, z - 1/2; (ii) x + 1/2, y + 1/2, -z + 3/2; (iii) x + 1/2, -y + 2, -z + 1; (iv) x + 1, -y + 3/2, z - 1/2; (v) x - 1/2, -y + 1, -z + 1; (vi) x + 1/2, y - 1/2, -z + 3/2; (b) (i) x, y + 1, z; (ii) -x + 1, -y + 1, -z + 1; (iii) -x, -y, -z + 1; (iv) -x + 1, -y, -z; (v) -x, -y + 1, -z + 1; (vi) x - 1, y, z; (vii) x - 1, y + 1, z + 1].

## supplementary crystallographic information

### Comment

The structure cis-bis(4-Aminopyridine)-bis(2,2'-bipyridine)-ruthenium(II) diperchlorate acetonitrile solvate (Duan *et al.*, 1999) have been reported previously. We present herein the crystal structure of the compound (I) with the hexafluorophosphate counterions.

Asymmetric units of complex (I) is shown in Fig. 1 and each Ru atom is coordinated to six nitrogen atoms from four ligand molecules. The compound (I) crystallizes in the noncentrosymmetric orthorhombic space group *P*2_1_cn, with one Ru(II) atom, two 2,2-bipyridine (bpy) ligands, two 4-aminopyridine (4Apy) and two PF_6_^-^ counterions in the asymmetric unit. The complex (I) is also present with one acetonitrile molecule in its asymmetric unit. Both compounds (I) and [Ru(C_10_H_8_N_2_)_2_(C_5_H_6_N_2_)_2_](ClO_4_)_2_ acetonitrile solvate [P-1 space group, cell parameters *a* =10.585 (2) Å, *b* = 11.615 (2) Å, *c* = 15.992 (3) Å, α = 104.44°, β = 99.76 (3)° and γ = 106.27°; Duan *et al.*, 1999] are isostructural: they crystallize in the centrosymmetric triclinic space group with very similar cell parameters and crystal packing features. Their crystal assemblies are stabilized by van der Waals interactions and classical hydrogen bonds whose NH_2_ groups of both 4Apy ligands are donors to either fluorine atoms (or oxygens of perchlorate in the antecedent isostructure) of the counterion or nitrogen one of acetonitrile solvent (Fig. 2). Indeed, four N—H···F interactions give rise to an intermolecularly-locked assembly of two centrosymmetric complex molecules and two PF_6_ counterions (Fig. 2). Two of them are related by a centrosymmetry to two independent ones, namely, the N4—H4A···F4 and N4A—H4A1···F5 contacts. These contacts involve both 4Apy moieties of (I) and one of the two crystallographically independent PF_6_ units. Such an assembly can be considered as the building unit of both crystal architectures of (I) and the perchlorate analogue. However, in the former two oxygens of one perchlorate unit, which has occupancy sites of some oxygen atoms disordered over two positions, substitute for F4 and F5 fluorine atoms of (I). It is important to observe such a disorder as that reported for the perchlorate analogue can be related to this packing feature described above. In (I), the F4—P1—F5 angle is 89.7 (7)°, while tetrahedral geometry of the four oxygens around chlorine atom of the perchlorate counterion imposes O—Cl—O angles to be near to 109°. No disorder is found for the positions of the fluorine supramolecular functionalities in (I), which is in agreement with the favorable geometric orientation of both F4 and F5 atoms to accept the hydrogen bonding from two inversion-related coordination complexes. In contrast, the expected angle values for ClO_4_ are much more enlarged than that of the *cis*-fluorine atoms acting as hydrogen bonding acceptors in N4—H4A···F4 and N4A—H4A1···F5, which clearly reveals the tendency for disordering the positions of the perchlorate oxygens in order to favor geometrically the formation of such contacts since the same intermolecular arrangement is kept in (I) and in the perchlorate analogue. No coordinates of the hydrogen atoms is available for the former structure, and therefore more detailed comparisons concerning the oxygen fractions involved in the hydrogen bonding interactions can not be accurately performed. The connection between the building blocks occurs along the [011] direction by mean of hydrogen bonding donation from both complex units of the centrosymmetric assembly to the other crystallographically independent PF_6_ fragment and to the acetonitrile solvent through the N4—H4B···F6A and N4A—H4A2···N1S contacts, respectively. In addition, there is a non-classical hydrogen bonding between this PF_6_ counterion and the solvent molecule through the C2S—H1S···F6A contact. In fact, two centrosymmetric motifs made up of hydrogen-bonded PF_6_ and acetonitrile molecules can be understood as the connecting pieces between the building units, giving rise to infinite one-dimensional ribbons running parallel to the [011] direction. Geometric parameters of the classical hydrogen bonding interactions are shown in Table 1.

The complex shows the Ru atom bonded to two bpy ligands in a *cis* configuration with the two 4Apy ligands in the expected distorted-octahedral fashion. The *trans* N—Ru—N angles (mean values of 175 (1)° (I), deviate slightly from the ideal value of 180°. Corresponding *cis* angles show similar small deviations from 90°: 91 (7)°. The mean Ru—*N*(α-diimine) distance [2.057 (7) Å] is similar to that found in a related coordination complex with the [Ru(bpy)_3_)]^2+^ moiety (2.056 Å) (Stoyanov *et al.*, 2002).

In the complex (I), the α-diimine coordinated ligands are approximately planar with deviation from the least-square planes less than 2°. The two bpy ligands are nearly perpendicular, as indicated by the dihedral angle between their least square planes, 82.71 (8)° in (I). The pyridine subunits in each bpy ligand are distorted by 2.7 (2)° [bpy(1)] and 6.1 (2)° [bpy(2)].

The dihedral angles do not show any significant distortions in the structure of the complex to relieve the steric hindrance imposed by bpy ligand. Fig. 1 shows a very neat twisted location of the 4Apy ligands with respect to the planes of the bpy ligands. The dihedral angles between the least-squares planes calculated through 4Apy and bpy ligands are 85.9 (1)° between [4Apy(1) and bpy(1)] and [4Apy(2) and bpy(2)].

### Experimental

The compound (I) was synthesized from the corresponding aquo-complex (Bonneson *et al.*, 1983) *cis*-[Ru(α-diimine)_2_(OH_2_)_2_](PF_6_)_2_ [α-diimine = 2,2-bipyridine (bpy)] by reacting the latter with 4-Aminopyridine in 1:1 EtOH/H_2_O mixture under nitrogen atmosphere and for 8 h under reflux. A stoichiometric amount of ammonium hexafluorophosphate was added to precipitate the complex. Single crystals suitable for X-ray diffraction measurement were obtained after 10 days on slow evaporation of an acetonitrile solution at room temperature. Elemental analysis (%) for (I) RuC_30_H_32_N_8_P_2_F_12_O_2_: calculated: C, 38.84, N, 12.08; H, 3.48; found: C, 38.70; N, 11.80; H, 3.54.

### Refinement

The H atoms were located from the difference Fourier synthesis and refined using the riding model on their parent atoms with C—H = 0.93 Å for aromatic moieties or 0.96 Å for methyl group of acetonitrile, N—H = 0.86 Å and *U*_iso_(H) = 1.2*U*_eq_ for phenyl and amine H atoms or 1.5*U*_eq_ for methyl ones.

### Crystal data

**Table:**

[Ru(C_10_H_8_N_2_)_2_(C_5_H_6_N_2_)_2_](PF_6_)_2_·C_2_H_3_N	*Z* = 2
*M**_r_* = 932.67	*F*(000) = 936
Triclinic, *P*1	*D*_x_ = 1.671 Mg m^−^^3^
Hall symbol: -P 1	Mo *K*α radiation, λ = 0.71073 Å
*a* = 10.8290 (3) Å	Cell parameters from 14506 reflections
*b* = 11.8890 (3) Å	θ = 2.9–27.2°
*c* = 16.1020 (4) Å	µ = 0.61 mm^−^^1^
α = 104.073 (1)°	*T* = 298 K
β = 99.114 (2)°	Prism, red
γ = 107.761 (2)°	0.21 × 0.11 × 0.09 mm
*V* = 1853.32 (8) Å^3^	

### Data collection

**Table:**

Nonius KappaCCD diffractometer	6142 reflections with *I* > 2σ(*I*)
CCD scans	*R*_int_ = 0.033
Absorption correction: multi-scan (Blessing, 1995)	θ_max_ = 27.2°, θ_min_ = 2.9°
*T*_min_ = 0.935, *T*_max_ = 0.950	*h* = −13→12
14506 measured reflections	*k* = −14→14
7906 independent reflections	*l* = −20→20

### Refinement

**Table:**

Refinement on *F*^2^	0 restraints
Least-squares matrix: full	H-atom parameters constrained
*R*[*F*^2^ > 2σ(*F*^2^)] = 0.053	*w* = 1/[σ^2^(*F*_o_^2^) + (0.081*P*)^2^ + 1.5061*P*] where *P* = (*F*_o_^2^ + 2*F*_c_^2^)/3
*wR*(*F*^2^) = 0.148	(Δ/σ)_max_ < 0.001
*S* = 1.06	Δρ_max_ = 0.90 e Å^−^^3^
7906 reflections	Δρ_min_ = −0.58 e Å^−^^3^
506 parameters	

### Fractional atomic coordinates and isotropic or equivalent isotropic displacement parameters (Å^2^)

**Table:**

	*x*	*y*	*z*	*U*_iso_*/*U*_eq_	
Ru	0.31099 (3)	0.67417 (3)	0.268302 (19)	0.03972 (12)	
N1	0.3873 (3)	0.8658 (3)	0.3076 (2)	0.0457 (7)	
N2	0.4801 (3)	0.7109 (3)	0.3647 (2)	0.0473 (7)	
N2A	0.4008 (3)	0.6761 (3)	0.1657 (2)	0.0444 (7)	
N1A	0.1542 (3)	0.6540 (3)	0.1681 (2)	0.0428 (7)	
N3	0.2084 (3)	0.6776 (3)	0.3689 (2)	0.0427 (7)	
N4	0.0286 (5)	0.6996 (5)	0.5798 (3)	0.0827 (13)	
H4A	−0.0134	0.7501	0.5909	0.099*	
H4B	0.0333	0.6524	0.6122	0.099*	
N3A	0.2449 (3)	0.4776 (3)	0.2282 (2)	0.0420 (7)	
N4A	0.0979 (5)	0.0921 (4)	0.1192 (3)	0.0834 (14)	
H4A1	0.017	0.049	0.1171	0.1*	
H4A2	0.1488	0.0558	0.098	0.1*	
C1	0.3343 (5)	0.9383 (4)	0.2738 (3)	0.0588 (11)	
H1	0.2531	0.9009	0.2312	0.071*	
C2	0.3946 (6)	1.0650 (4)	0.2994 (4)	0.0705 (13)	
H2	0.3546	1.1124	0.2748	0.085*	
C3	0.5142 (6)	1.1206 (5)	0.3616 (4)	0.0785 (15)	
H3	0.558	1.2063	0.3791	0.094*	
C4	0.5690 (5)	1.0480 (5)	0.3980 (4)	0.0727 (14)	
H4	0.6494	1.0846	0.4415	0.087*	
C5	0.5051 (4)	0.9214 (4)	0.3701 (3)	0.0510 (10)	
C6	0.5564 (4)	0.8353 (4)	0.4037 (3)	0.0526 (10)	
C7	0.6732 (5)	0.8742 (5)	0.4703 (3)	0.0750 (14)	
H7	0.7231	0.9584	0.4966	0.09*	
C8	0.7141 (5)	0.7874 (6)	0.4966 (4)	0.0846 (17)	
H8	0.7926	0.8119	0.5404	0.102*	
C9	0.6390 (6)	0.6658 (6)	0.4582 (4)	0.0856 (18)	
H9	0.6647	0.6058	0.476	0.103*	
C10	0.5253 (5)	0.6317 (5)	0.3930 (3)	0.0693 (13)	
H10	0.4763	0.5473	0.3668	0.083*	
C10A	0.5286 (4)	0.6843 (4)	0.1691 (3)	0.0577 (11)	
H10A	0.5829	0.6892	0.2217	0.069*	
C9A	0.5807 (5)	0.6855 (5)	0.0966 (4)	0.0733 (14)	
H9A	0.6694	0.6921	0.1008	0.088*	
C8A	0.5032 (6)	0.6772 (6)	0.0192 (4)	0.0804 (15)	
H8A	0.537	0.6753	−0.0305	0.097*	
C7A	0.3718 (5)	0.6716 (5)	0.0148 (3)	0.0713 (14)	
H7A	0.3175	0.6678	−0.0374	0.086*	
C6A	0.3240 (4)	0.6716 (4)	0.0888 (3)	0.0497 (9)	
C5A	0.1874 (4)	0.6646 (4)	0.0911 (3)	0.0475 (9)	
C4A	0.0962 (5)	0.6651 (5)	0.0210 (3)	0.0609 (11)	
H4A3	0.1212	0.6742	−0.0303	0.073*	
C3A	−0.0327 (5)	0.6521 (5)	0.0278 (3)	0.0712 (14)	
H3A	−0.0948	0.6544	−0.0182	0.085*	
C2A	−0.0675 (4)	0.6356 (5)	0.1033 (3)	0.0635 (12)	
H2A	−0.1548	0.6228	0.1082	0.076*	
C1A	0.0277 (4)	0.6383 (4)	0.1717 (3)	0.0506 (9)	
H1A	0.0031	0.6288	0.223	0.061*	
C11	0.2122 (5)	0.6049 (4)	0.4215 (3)	0.0607 (11)	
H11	0.2557	0.5487	0.4094	0.073*	
C12	0.1551 (5)	0.6104 (5)	0.4914 (3)	0.0642 (12)	
H12	0.1604	0.559	0.5261	0.077*	
C13	0.0881 (4)	0.6942 (4)	0.5106 (3)	0.0564 (10)	
C14	0.0837 (4)	0.7662 (4)	0.4576 (3)	0.0595 (11)	
H14	0.04	0.8226	0.4677	0.071*	
C15	0.1447 (4)	0.7554 (4)	0.3884 (3)	0.0558 (10)	
H15	0.1406	0.8063	0.3532	0.067*	
C11A	0.3199 (4)	0.4140 (4)	0.1952 (3)	0.0501 (9)	
H11A	0.4075	0.4593	0.1968	0.06*	
C12A	0.2760 (4)	0.2877 (4)	0.1598 (3)	0.0533 (10)	
H12A	0.3332	0.2497	0.1387	0.064*	
C13A	0.1447 (4)	0.2160 (4)	0.1555 (3)	0.0544 (10)	
C14A	0.0678 (4)	0.2799 (4)	0.1908 (3)	0.0558 (10)	
H14A	−0.0194	0.2362	0.1912	0.067*	
C15A	0.1186 (4)	0.4061 (4)	0.2248 (3)	0.0485 (9)	
H15A	0.0634	0.4457	0.247	0.058*	
P1	0.07844 (14)	0.04876 (12)	0.70888 (9)	0.0660 (3)	
P1A	0.71818 (12)	0.40171 (12)	0.25261 (9)	0.0604 (3)	
F1	−0.0516 (5)	0.0689 (5)	0.7229 (5)	0.158 (2)	
F2	0.1439 (5)	0.1892 (3)	0.7154 (3)	0.1230 (15)	
F3	0.2117 (6)	0.0324 (7)	0.6988 (4)	0.172 (3)	
F4	0.0089 (7)	−0.0907 (4)	0.7029 (3)	0.160 (2)	
F5	0.1258 (5)	0.0774 (5)	0.8116 (3)	0.1387 (18)	
F6	0.0318 (6)	0.0146 (5)	0.6062 (3)	0.148 (2)	
F6A	0.7482 (4)	0.2948 (4)	0.2846 (3)	0.1114 (12)	
F4A	0.6327 (5)	0.4106 (5)	0.3225 (3)	0.1294 (15)	
F1A	0.6852 (5)	0.5050 (4)	0.2214 (4)	0.1450 (19)	
F2A	0.8037 (3)	0.3839 (5)	0.1829 (2)	0.1177 (15)	
F5A	0.5890 (3)	0.3042 (4)	0.1804 (2)	0.1011 (11)	
F3A	0.8512 (4)	0.4961 (4)	0.3229 (3)	0.1229 (15)	
C2S	0.6126 (9)	0.0190 (7)	0.1288 (5)	0.112 (2)	
H3S	0.5892	−0.0603	0.1389	0.168*	
H2S	0.5333	0.0381	0.1152	0.168*	
H1S	0.6742	0.0818	0.181	0.168*	
C1S	0.6767 (9)	0.0151 (7)	0.0534 (8)	0.124 (3)	
N1S	0.7190 (10)	0.0059 (9)	−0.0082 (8)	0.189 (5)	

### Atomic displacement parameters (Å^2^)

**Table:**

	*U*^11^	*U*^22^	*U*^33^	*U*^12^	*U*^13^	*U*^23^
Ru	0.03023 (16)	0.03983 (18)	0.04288 (19)	0.01056 (12)	0.00455 (11)	0.00750 (12)
N1	0.0382 (16)	0.0436 (17)	0.0499 (18)	0.0110 (14)	0.0133 (14)	0.0083 (14)
N2	0.0422 (17)	0.0511 (19)	0.0462 (18)	0.0193 (15)	0.0089 (14)	0.0085 (15)
N2A	0.0359 (16)	0.0404 (16)	0.0518 (18)	0.0096 (13)	0.0121 (14)	0.0099 (14)
N1A	0.0353 (15)	0.0386 (16)	0.0456 (17)	0.0104 (13)	0.0058 (13)	0.0038 (13)
N3	0.0407 (16)	0.0438 (17)	0.0430 (17)	0.0165 (14)	0.0083 (13)	0.0117 (13)
N4	0.097 (3)	0.095 (3)	0.070 (3)	0.043 (3)	0.039 (3)	0.029 (2)
N3A	0.0344 (15)	0.0393 (16)	0.0504 (18)	0.0122 (13)	0.0104 (13)	0.0118 (13)
N4A	0.080 (3)	0.043 (2)	0.116 (4)	0.010 (2)	0.043 (3)	0.010 (2)
C1	0.057 (3)	0.052 (2)	0.066 (3)	0.017 (2)	0.014 (2)	0.019 (2)
C2	0.088 (4)	0.047 (3)	0.079 (3)	0.021 (2)	0.028 (3)	0.023 (2)
C3	0.080 (4)	0.045 (3)	0.093 (4)	0.001 (2)	0.025 (3)	0.014 (3)
C4	0.051 (3)	0.060 (3)	0.078 (3)	−0.002 (2)	0.009 (2)	0.003 (2)
C5	0.0359 (19)	0.054 (2)	0.050 (2)	0.0053 (17)	0.0118 (16)	0.0071 (18)
C6	0.0354 (19)	0.062 (3)	0.046 (2)	0.0087 (18)	0.0082 (16)	0.0034 (19)
C7	0.044 (2)	0.085 (4)	0.066 (3)	0.009 (2)	−0.004 (2)	0.001 (3)
C8	0.057 (3)	0.105 (5)	0.069 (3)	0.035 (3)	−0.016 (2)	0.000 (3)
C9	0.079 (4)	0.105 (5)	0.075 (3)	0.059 (4)	−0.006 (3)	0.014 (3)
C10	0.064 (3)	0.073 (3)	0.065 (3)	0.036 (3)	−0.004 (2)	0.010 (2)
C10A	0.039 (2)	0.062 (3)	0.067 (3)	0.0166 (19)	0.0152 (19)	0.012 (2)
C9A	0.050 (3)	0.085 (4)	0.092 (4)	0.027 (2)	0.033 (3)	0.024 (3)
C8A	0.072 (3)	0.104 (4)	0.070 (3)	0.032 (3)	0.035 (3)	0.024 (3)
C7A	0.060 (3)	0.092 (4)	0.054 (3)	0.017 (3)	0.020 (2)	0.018 (2)
C6A	0.041 (2)	0.051 (2)	0.048 (2)	0.0099 (17)	0.0080 (17)	0.0097 (18)
C5A	0.042 (2)	0.048 (2)	0.045 (2)	0.0130 (17)	0.0066 (16)	0.0086 (17)
C4A	0.055 (3)	0.072 (3)	0.050 (2)	0.019 (2)	0.0047 (19)	0.020 (2)
C3A	0.059 (3)	0.090 (4)	0.060 (3)	0.034 (3)	−0.007 (2)	0.018 (3)
C2A	0.041 (2)	0.075 (3)	0.066 (3)	0.027 (2)	0.001 (2)	0.008 (2)
C1A	0.037 (2)	0.055 (2)	0.055 (2)	0.0171 (17)	0.0059 (17)	0.0096 (19)
C11	0.069 (3)	0.064 (3)	0.050 (2)	0.028 (2)	0.012 (2)	0.015 (2)
C12	0.074 (3)	0.065 (3)	0.052 (3)	0.024 (2)	0.012 (2)	0.020 (2)
C13	0.055 (2)	0.056 (2)	0.048 (2)	0.015 (2)	0.0139 (19)	0.0045 (19)
C14	0.055 (3)	0.055 (3)	0.061 (3)	0.022 (2)	0.007 (2)	0.006 (2)
C15	0.052 (2)	0.055 (2)	0.055 (2)	0.019 (2)	0.0071 (19)	0.0100 (19)
C11A	0.0355 (19)	0.052 (2)	0.062 (2)	0.0144 (17)	0.0129 (17)	0.0172 (19)
C12A	0.049 (2)	0.048 (2)	0.065 (3)	0.0211 (19)	0.020 (2)	0.0151 (19)
C13A	0.055 (2)	0.047 (2)	0.058 (2)	0.0147 (19)	0.0173 (19)	0.0139 (19)
C14A	0.042 (2)	0.053 (2)	0.064 (3)	0.0072 (18)	0.0164 (19)	0.013 (2)
C15A	0.039 (2)	0.051 (2)	0.056 (2)	0.0157 (17)	0.0149 (17)	0.0159 (18)
P1	0.0642 (8)	0.0582 (7)	0.0740 (8)	0.0224 (6)	0.0170 (6)	0.0175 (6)
P1A	0.0514 (6)	0.0643 (7)	0.0654 (7)	0.0217 (6)	0.0145 (5)	0.0186 (6)
F1	0.099 (3)	0.173 (5)	0.260 (7)	0.081 (3)	0.076 (4)	0.105 (5)
F2	0.141 (4)	0.070 (2)	0.132 (3)	0.005 (2)	0.034 (3)	0.027 (2)
F3	0.157 (4)	0.269 (7)	0.213 (6)	0.147 (5)	0.121 (4)	0.149 (5)
F4	0.271 (7)	0.063 (2)	0.164 (4)	0.047 (3)	0.119 (5)	0.042 (2)
F5	0.119 (3)	0.183 (5)	0.088 (3)	0.015 (3)	0.015 (2)	0.054 (3)
F6	0.181 (5)	0.123 (4)	0.082 (3)	−0.003 (3)	0.002 (3)	0.021 (2)
F6A	0.124 (3)	0.105 (3)	0.116 (3)	0.056 (2)	0.015 (2)	0.044 (2)
F4A	0.151 (4)	0.158 (4)	0.112 (3)	0.080 (3)	0.075 (3)	0.042 (3)
F1A	0.171 (5)	0.104 (3)	0.177 (5)	0.064 (3)	0.015 (4)	0.074 (3)
F2A	0.0598 (19)	0.208 (5)	0.081 (2)	0.044 (2)	0.0248 (17)	0.038 (3)
F5A	0.0564 (18)	0.117 (3)	0.102 (3)	0.0137 (18)	0.0072 (17)	0.016 (2)
F3A	0.107 (3)	0.113 (3)	0.086 (2)	−0.016 (2)	−0.014 (2)	0.017 (2)
C2S	0.136 (7)	0.096 (5)	0.105 (5)	0.060 (5)	0.026 (5)	0.012 (4)
C1S	0.100 (6)	0.089 (5)	0.161 (9)	0.049 (5)	−0.011 (5)	0.007 (5)
N1S	0.179 (9)	0.139 (7)	0.257 (12)	0.057 (6)	0.133 (9)	0.027 (7)

### Geometric parameters (Å, º)

**Table:**

Ru—N2A	2.047 (3)	C8A—H8A	0.93
Ru—N2	2.059 (3)	C7A—C6A	1.372 (6)
Ru—N1A	2.058 (3)	C7A—H7A	0.93
Ru—N1	2.063 (3)	C6A—C5A	1.463 (5)
Ru—N3	2.104 (3)	C5A—C4A	1.380 (6)
Ru—N3A	2.119 (3)	C4A—C3A	1.381 (7)
N1—C1	1.341 (5)	C4A—H4A3	0.93
N1—C5	1.354 (5)	C3A—C2A	1.370 (7)
N2—C10	1.324 (6)	C3A—H3A	0.93
N2—C6	1.379 (5)	C2A—C1A	1.371 (6)
N2A—C10A	1.349 (5)	C2A—H2A	0.93
N2A—C6A	1.358 (5)	C1A—H1A	0.93
N1A—C1A	1.338 (5)	C11—C12	1.365 (7)
N1A—C5A	1.369 (5)	C11—H11	0.93
N3—C15	1.321 (5)	C12—C13	1.406 (7)
N3—C11	1.353 (6)	C12—H12	0.93
N4—C13	1.372 (6)	C13—C14	1.353 (7)
N4—H4A	0.86	C14—C15	1.382 (6)
N4—H4B	0.86	C14—H14	0.93
N3A—C11A	1.352 (5)	C15—H15	0.93
N3A—C15A	1.357 (5)	C11A—C12A	1.367 (6)
N4A—C13A	1.342 (6)	C11A—H11A	0.93
N4A—H4A1	0.86	C12A—C13A	1.398 (6)
N4A—H4A2	0.86	C12A—H12A	0.93
C1—C2	1.371 (6)	C13A—C14A	1.386 (6)
C1—H1	0.93	C14A—C15A	1.362 (6)
C2—C3	1.365 (8)	C14A—H14A	0.93
C2—H2	0.93	C15A—H15A	0.93
C3—C4	1.374 (8)	P1—F1	1.540 (4)
C3—H3	0.93	P1—F3	1.542 (5)
C4—C5	1.372 (6)	P1—F6	1.561 (4)
C4—H4	0.93	P1—F5	1.570 (4)
C5—C6	1.474 (6)	P1—F4	1.570 (4)
C6—C7	1.391 (6)	P1—F2	1.572 (4)
C7—C8	1.369 (8)	P1A—F1A	1.548 (4)
C7—H7	0.93	P1A—F4A	1.570 (4)
C8—C9	1.349 (9)	P1A—F5A	1.579 (3)
C8—H8	0.93	P1A—F3A	1.580 (4)
C9—C10	1.364 (7)	P1A—F2A	1.581 (4)
C9—H9	0.93	P1A—F6A	1.582 (4)
C10—H10	0.93	C2S—C1S	1.489 (13)
C10A—C9A	1.376 (7)	C2S—H3S	0.96
C10A—H10A	0.93	C2S—H2S	0.96
C9A—C8A	1.350 (8)	C2S—H1S	0.96
C9A—H9A	0.93	C1S—N1S	1.152 (13)
C8A—C7A	1.392 (7)		
			
N2A—Ru—N2	96.95 (13)	C7A—C6A—C5A	123.4 (4)
N2A—Ru—N1A	78.94 (12)	N1A—C5A—C4A	121.6 (4)
N2—Ru—N1A	173.63 (13)	N1A—C5A—C6A	114.8 (3)
N2A—Ru—N1	87.84 (12)	C4A—C5A—C6A	123.5 (4)
N2—Ru—N1	79.24 (13)	C5A—C4A—C3A	119.4 (4)
N1A—Ru—N1	95.66 (12)	C5A—C4A—H4A3	120.3
N2A—Ru—N3	175.56 (12)	C3A—C4A—H4A3	120.3
N2—Ru—N3	86.83 (12)	C2A—C3A—C4A	118.9 (4)
N1A—Ru—N3	97.09 (12)	C2A—C3A—H3A	120.6
N1—Ru—N3	90.57 (12)	C4A—C3A—H3A	120.6
N2A—Ru—N3A	90.07 (12)	C3A—C2A—C1A	119.3 (4)
N2—Ru—N3A	98.16 (12)	C3A—C2A—H2A	120.3
N1A—Ru—N3A	86.77 (12)	C1A—C2A—H2A	120.3
N1—Ru—N3A	176.43 (11)	N1A—C1A—C2A	123.2 (4)
N3—Ru—N3A	91.72 (12)	N1A—C1A—H1A	118.4
C1—N1—C5	117.9 (4)	C2A—C1A—H1A	118.4
C1—N1—Ru	126.0 (3)	N3—C11—C12	123.0 (4)
C5—N1—Ru	116.0 (3)	N3—C11—H11	118.5
C10—N2—C6	116.5 (4)	C12—C11—H11	118.5
C10—N2—Ru	128.8 (3)	C11—C12—C13	119.5 (4)
C6—N2—Ru	114.7 (3)	C11—C12—H12	120.2
C10A—N2A—C6A	118.3 (4)	C13—C12—H12	120.2
C10A—N2A—Ru	125.7 (3)	C14—C13—N4	122.4 (5)
C6A—N2A—Ru	116.0 (3)	C14—C13—C12	117.4 (4)
C1A—N1A—C5A	117.4 (3)	N4—C13—C12	120.2 (5)
C1A—N1A—Ru	127.5 (3)	C13—C14—C15	119.5 (4)
C5A—N1A—Ru	114.9 (2)	C13—C14—H14	120.3
C15—N3—C11	116.2 (4)	C15—C14—H14	120.3
C15—N3—Ru	122.6 (3)	N3—C15—C14	124.4 (4)
C11—N3—Ru	121.0 (3)	N3—C15—H15	117.8
C13—N4—H4A	120	C14—C15—H15	117.8
C13—N4—H4B	120	N3A—C11A—C12A	124.6 (4)
H4A—N4—H4B	120	N3A—C11A—H11A	117.7
C11A—N3A—C15A	114.8 (3)	C12A—C11A—H11A	117.7
C11A—N3A—Ru	122.7 (3)	C11A—C12A—C13A	119.5 (4)
C15A—N3A—Ru	122.1 (3)	C11A—C12A—H12A	120.2
C13A—N4A—H4A1	120	C13A—C12A—H12A	120.2
C13A—N4A—H4A2	120	N4A—C13A—C14A	122.8 (4)
H4A1—N4A—H4A2	120	N4A—C13A—C12A	120.9 (4)
N1—C1—C2	122.9 (5)	C14A—C13A—C12A	116.4 (4)
N1—C1—H1	118.5	C15A—C14A—C13A	120.6 (4)
C2—C1—H1	118.5	C15A—C14A—H14A	119.7
C3—C2—C1	118.9 (5)	C13A—C14A—H14A	119.7
C3—C2—H2	120.5	N3A—C15A—C14A	124.0 (4)
C1—C2—H2	120.5	N3A—C15A—H15A	118
C2—C3—C4	119.0 (5)	C14A—C15A—H15A	118
C2—C3—H3	120.5	F1—P1—F3	177.6 (4)
C4—C3—H3	120.5	F1—P1—F6	93.1 (4)
C5—C4—C3	120.0 (5)	F3—P1—F6	89.1 (4)
C5—C4—H4	120	F1—P1—F5	88.2 (3)
C3—C4—H4	120	F3—P1—F5	89.7 (3)
N1—C5—C4	121.2 (4)	F6—P1—F5	177.5 (3)
N1—C5—C6	114.5 (3)	F1—P1—F4	88.0 (3)
C4—C5—C6	124.2 (4)	F3—P1—F4	93.1 (4)
N2—C6—C7	121.2 (4)	F6—P1—F4	90.4 (3)
N2—C6—C5	115.4 (3)	F5—P1—F4	87.5 (3)
C7—C6—C5	123.4 (4)	F1—P1—F2	90.2 (3)
C8—C7—C6	119.3 (5)	F3—P1—F2	88.7 (3)
C8—C7—H7	120.3	F6—P1—F2	89.7 (2)
C6—C7—H7	120.3	F5—P1—F2	92.4 (3)
C9—C8—C7	119.2 (5)	F4—P1—F2	178.2 (3)
C9—C8—H8	120.4	F1A—P1A—F4A	91.9 (3)
C7—C8—H8	120.4	F1A—P1A—F5A	88.0 (3)
C8—C9—C10	119.6 (5)	F4A—P1A—F5A	89.8 (2)
C8—C9—H9	120.2	F1A—P1A—F3A	93.4 (3)
C10—C9—H9	120.2	F4A—P1A—F3A	92.3 (3)
N2—C10—C9	124.2 (5)	F5A—P1A—F3A	177.5 (3)
N2—C10—H10	117.9	F1A—P1A—F2A	91.8 (3)
C9—C10—H10	117.9	F4A—P1A—F2A	176.1 (3)
N2A—C10A—C9A	121.7 (4)	F5A—P1A—F2A	89.1 (2)
N2A—C10A—H10A	119.2	F3A—P1A—F2A	88.7 (2)
C9A—C10A—H10A	119.2	F1A—P1A—F6A	178.6 (3)
C8A—C9A—C10A	120.1 (5)	F4A—P1A—F6A	87.1 (3)
C8A—C9A—H9A	120	F5A—P1A—F6A	91.1 (2)
C10A—C9A—H9A	120	F3A—P1A—F6A	87.6 (2)
C9A—C8A—C7A	119.2 (5)	F2A—P1A—F6A	89.2 (2)
C9A—C8A—H8A	120.4	C1S—C2S—H3S	109.5
C7A—C8A—H8A	120.4	C1S—C2S—H2S	109.5
C6A—C7A—C8A	118.9 (5)	H3S—C2S—H2S	109.5
C6A—C7A—H7A	120.6	C1S—C2S—H1S	109.5
C8A—C7A—H7A	120.6	H3S—C2S—H1S	109.5
N2A—C6A—C7A	121.8 (4)	H2S—C2S—H1S	109.5
N2A—C6A—C5A	114.8 (4)	N1S—C1S—C2S	175.7 (10)
			
N2A—Ru—N1—C1	81.4 (3)	Ru—N2—C6—C7	179.7 (3)
N2—Ru—N1—C1	178.9 (4)	C10—N2—C6—C5	179.1 (4)
N1A—Ru—N1—C1	2.8 (3)	Ru—N2—C6—C5	−0.2 (4)
N3—Ru—N1—C1	−94.4 (3)	N1—C5—C6—N2	2.2 (5)
N2A—Ru—N1—C5	−95.1 (3)	C4—C5—C6—N2	−177.4 (4)
N2—Ru—N1—C5	2.4 (3)	N1—C5—C6—C7	−177.6 (4)
N1A—Ru—N1—C5	−173.7 (3)	C4—C5—C6—C7	2.7 (7)
N3—Ru—N1—C5	89.1 (3)	N2—C6—C7—C8	0.9 (7)
N2A—Ru—N2—C10	−93.8 (4)	C5—C6—C7—C8	−179.3 (5)
N1—Ru—N2—C10	179.7 (4)	C6—C7—C8—C9	−0.9 (9)
N3—Ru—N2—C10	88.5 (4)	C7—C8—C9—C10	1.0 (10)
N3A—Ru—N2—C10	−2.7 (4)	C6—N2—C10—C9	1.3 (7)
N2A—Ru—N2—C6	85.3 (3)	Ru—N2—C10—C9	−179.6 (4)
N1—Ru—N2—C6	−1.1 (3)	C8—C9—C10—N2	−1.3 (9)
N3—Ru—N2—C6	−92.3 (3)	C6A—N2A—C10A—C9A	−1.4 (6)
N3A—Ru—N2—C6	176.4 (3)	Ru—N2A—C10A—C9A	−179.7 (4)
N2—Ru—N2A—C10A	7.0 (4)	N2A—C10A—C9A—C8A	−0.6 (8)
N1A—Ru—N2A—C10A	−177.9 (4)	C10A—C9A—C8A—C7A	2.1 (9)
N1—Ru—N2A—C10A	85.9 (3)	C9A—C8A—C7A—C6A	−1.5 (9)
N3A—Ru—N2A—C10A	−91.2 (3)	C10A—N2A—C6A—C7A	2.0 (6)
N2—Ru—N2A—C6A	−171.3 (3)	Ru—N2A—C6A—C7A	−179.6 (4)
N1A—Ru—N2A—C6A	3.8 (3)	C10A—N2A—C6A—C5A	−179.2 (4)
N1—Ru—N2A—C6A	−92.4 (3)	Ru—N2A—C6A—C5A	−0.7 (4)
N3A—Ru—N2A—C6A	90.5 (3)	C8A—C7A—C6A—N2A	−0.5 (8)
N2A—Ru—N1A—C1A	177.3 (3)	C8A—C7A—C6A—C5A	−179.3 (5)
N1—Ru—N1A—C1A	−96.0 (3)	C1A—N1A—C5A—C4A	3.0 (6)
N3—Ru—N1A—C1A	−4.7 (3)	Ru—N1A—C5A—C4A	−173.7 (3)
N3A—Ru—N1A—C1A	86.6 (3)	C1A—N1A—C5A—C6A	−175.4 (4)
N2A—Ru—N1A—C5A	−6.4 (3)	Ru—N1A—C5A—C6A	7.9 (4)
N1—Ru—N1A—C5A	80.3 (3)	N2A—C6A—C5A—N1A	−4.7 (5)
N3—Ru—N1A—C5A	171.6 (3)	C7A—C6A—C5A—N1A	174.1 (4)
N3A—Ru—N1A—C5A	−97.1 (3)	N2A—C6A—C5A—C4A	176.9 (4)
N2—Ru—N3—C15	121.1 (3)	C7A—C6A—C5A—C4A	−4.3 (7)
N1A—Ru—N3—C15	−53.9 (3)	N1A—C5A—C4A—C3A	−1.4 (7)
N1—Ru—N3—C15	41.9 (3)	C6A—C5A—C4A—C3A	176.9 (4)
N3A—Ru—N3—C15	−140.8 (3)	C5A—C4A—C3A—C2A	−1.7 (8)
N2—Ru—N3—C11	−54.3 (3)	C4A—C3A—C2A—C1A	3.0 (8)
N1A—Ru—N3—C11	130.7 (3)	C5A—N1A—C1A—C2A	−1.7 (6)
N1—Ru—N3—C11	−133.5 (3)	Ru—N1A—C1A—C2A	174.6 (3)
N3A—Ru—N3—C11	43.8 (3)	C3A—C2A—C1A—N1A	−1.3 (7)
N2A—Ru—N3A—C11A	37.0 (3)	C15—N3—C11—C12	−0.4 (6)
N2—Ru—N3A—C11A	−60.1 (3)	Ru—N3—C11—C12	175.3 (4)
N1A—Ru—N3A—C11A	115.9 (3)	N3—C11—C12—C13	0.4 (7)
N3—Ru—N3A—C11A	−147.1 (3)	C11—C12—C13—C14	0.0 (7)
N2A—Ru—N3A—C15A	−135.8 (3)	C11—C12—C13—N4	179.1 (4)
N2—Ru—N3A—C15A	127.2 (3)	N4—C13—C14—C15	−179.4 (4)
N1A—Ru—N3A—C15A	−56.9 (3)	C12—C13—C14—C15	−0.3 (7)
N3—Ru—N3A—C15A	40.1 (3)	C11—N3—C15—C14	0.1 (6)
C5—N1—C1—C2	0.5 (6)	Ru—N3—C15—C14	−175.5 (3)
Ru—N1—C1—C2	−176.0 (4)	C13—C14—C15—N3	0.3 (7)
N1—C1—C2—C3	0.4 (8)	C15A—N3A—C11A—C12A	0.6 (6)
C1—C2—C3—C4	−1.4 (8)	Ru—N3A—C11A—C12A	−172.6 (3)
C2—C3—C4—C5	1.6 (8)	N3A—C11A—C12A—C13A	0.5 (7)
C1—N1—C5—C4	−0.3 (6)	C11A—C12A—C13A—N4A	178.4 (5)
Ru—N1—C5—C4	176.5 (3)	C11A—C12A—C13A—C14A	−1.8 (7)
C1—N1—C5—C6	−180.0 (4)	N4A—C13A—C14A—C15A	−178.2 (5)
Ru—N1—C5—C6	−3.2 (4)	C12A—C13A—C14A—C15A	2.1 (7)
C3—C4—C5—N1	−0.7 (7)	C11A—N3A—C15A—C14A	−0.4 (6)
C3—C4—C5—C6	178.9 (5)	Ru—N3A—C15A—C14A	172.9 (3)
C10—N2—C6—C7	−1.1 (6)	C13A—C14A—C15A—N3A	−1.0 (7)

### Hydrogen-bond geometry (Å, º)

**Table:**

*D*—H···*A*	*D*—H	H···*A*	*D*···*A*	*D*—H···*A*
N4—H4*A*···F4^i^	0.86	2.19	2.871 (8)	135
N4—H4*B*···F6*A*^ii^	0.86	2.47	2.962 (7)	117
N4*A*—H4*A*1···F5^iii^	0.86	2.44	3.184 (8)	145
N4*A*—H4*A*2···N1*S*^iv^	0.86	2.34	3.162 (13)	161

Symmetry codes: (i) *x*, *y*+1, *z*; (ii) −*x*+1, −*y*+1, −*z*+1; (iii) −*x*, −*y*, −*z*+1; (iv) −*x*+1, −*y*, −*z*.
